# The ORION study protocol: impact of prior intraperitoneal versus extraperitoneal mesh placement on subsequent minimally invasive abdominal reoperations

**DOI:** 10.3389/jaws.2026.17325

**Published:** 2026-09-18

**Authors:** Elisa Pelfini, Sara Lauricella, Antonella Nisi, Gianlorenzo Dionigi, Francesco Brucchi

**Affiliations:** 1 General Surgery Residency Program, University of Milan, Milan, Italy; 2 Policlinico San Pietro, Unit of Colon Proctology and Pelviperineology, Bergamo, Italy; 3 Division of General Surgery, IRCCS Istituto Auxologico Italiano, Milan, Italy; 4 Department of Pathophysiology and Transplantation, University of Milan, Milan, Italy

**Keywords:** hernia recurrence, mesh, multicentric prospective study, pain, ventral hernia repair

## Abstract

**Background:**

Ventral hernia repair is among the most common surgical operations worldwide. Mesh can be positioned in different anatomical planes; extraperitoneal placement is gaining popularity, whereas intraperitoneal placement is linked to long-term complications. Evidence comparing the long-term outcomes of intraperitoneal versus extraperitoneal placement remains limited.

**Objective:**

This study addresses that gap, also examining the management of recurrences and the potential neurological sequelae of surgery.

**Methods:**

The ORION study is a prospective, multicentre, observational study involving surgical centres across Europe. The study is addressed to patients who have previously undergone minimally invasive mesh-based ventral hernia repair, with either intraperitoneal or extraperitoneal mesh placement, and who are now undergoing minimally invasive abdominal surgery—either in an elective or emergency setting—for a hernia recurrence (Cohort B) or for other indications (Cohort A). Data will be collected between 1 March 2026 and 28 February 2027. Follow-up assessments at 30 and 90 days are planned only for patients enrolled in Cohort B.

**Discussion:**

The ORION study will provide data on the intraoperative consequences of prior intraperitoneal versus extraperitoneal mesh placement at subsequent minimally invasive abdominal surgery, and will describe recurrence patterns and management among patients reoperated for recurrent ventral/incisional hernia.

**Clinical Trial Registration:**

Identifier NCT07384962.

## Introduction

Ventral hernia repair is amongst the most common surgical operations performed worldwide [1]. Large meta-analyses have established the effectiveness of minimally-invasive (MIS) incisional and ventral hernia repair, with some literature indicating that it offers superior outcomes in terms of postoperative complications and may be beneficial also in terms of recurrence rates when compared to open surgery [2–5].

In MIS repair, the mesh can be positioned in various anatomical planes, including intraperitoneal, preperitoneal, retro-muscular, and onlay (pre-fascial). Particularly, techniques involving mesh placement outside the peritoneal cavity are gaining popularity as alternatives to intraperitoneal mesh repair [6] due to increased short-term postoperative pain (due to the use of tackers [7]) and early recurrence (due to mesh migration [8]). However, the most appropriate mesh position still remains controversial [9].

Intraperitoneal mesh placement is linked to long-term complications such as bowel entrapment and intra-abdominal adhesions, which can cause pain or discomfort [10, 11]. However, most studies focus on hernia-related outcomes like recurrence and infection, potentially underreporting long-term complications from adhesions, such as bowel obstruction or pain. Adhesions between the bowel and mesh are common in imaging and surgical revisions in these patients [12]. Animal studies show that even composite meshes provoke adhesion formation in the intraperitoneal space [13, 14].

Despite existing knowledge, there is a significant lack of evidence regarding the long-term outcomes of intraperitoneal versus extraperitoneal mesh placement. To address this gap, the present study will be an international collaborative initiative aimed at collecting data from patients undergoing minimally-invasive abdominal procedures, who have previously undergone minimally-invasive ventral hernia repair with either intraperitoneal or extraperitoneal mesh placement.

This investigation will also focus on the management of ventral hernia recurrences and on the possible neurological sequelae of this surgical intervention—an area of increasing relevance, as chronic post-surgical pain and neuropathic sequelae following ventral hernia repair represent an under-recognized morbidity potentially influenced by mesh position and fixation method. The study will therefore compare the impact of the two different surgical strategies on postoperative complications and on the consequent need for specific neurological therapy.

### Main and secondary hypothesis

We hypothesise that prior intraperitoneal mesh placement is associated with a higher risk of intraoperative visceral injury during adhesiolysis at subsequent minimally invasive abdominal surgery than prior extraperitoneal placement (primary hypothesis). Secondarily, we hypothesise that intraperitoneal placement is associated with a greater burden of adhesion-related complications, longer adhesiolysis and a higher conversion rate, and that, among patients operated on for a recurrence, it is associated with higher rates of chronic post-surgical pain and neuropathic features at 90 days. The corresponding null hypotheses state that the two mesh positions do not differ with respect to these outcomes.

## Methods and design

### Study design

The ORION Study is a prospective, multicentre, observational study involving multiple surgical centers across Europe. The study will follow patients who have previously undergone minimally-invasive mesh-based ventral hernia repair (intraperitoneal or extraperitoneal placement) and are now scheduled to undergo a subsequent minimally-invasive abdominal surgery, between 1 March 2026 to 28 February 2027. Mini-teams of up to three collaborators will prospectively collect data at each participating centre. The project follows the principles of collaborative clinical research endorsed by the European Hernia Society (EHS) and involves voluntary participation of surgical centres across Europe. The study is conducted in accordance with the STROCSS statement for observational studies.

### Study setting

This prospective study will be carried out from March 2026 across Europe. The study is open to any European hospital that performs emergency and/or elective minimally-invasive abdominal surgery. An eligible hospital must collect consecutive patients undergoing minimally-invasive abdominal surgery who previously underwent intraperitoneal or extraperitoneal mesh placement for ventral hernia repair during the specified study period, following appropriate registration of the study according to local hospital regulations. There is no minimum number of patients per centre.

### Study population

We prospectively include all consecutive adult patients undergoing minimally invasive abdominal surgery after a previous minimally-invasive ventral hernia repair with mesh (intraperitoneal or extraperitoneal), irrespective of the current indication (e.g., cholecystectomy, colorectal, gynecologic, or hernia surgery) (Figure 1). This design captures the real-world risk of adhesiolysis-related visceral injury across procedures.

**FIGURE 1:**
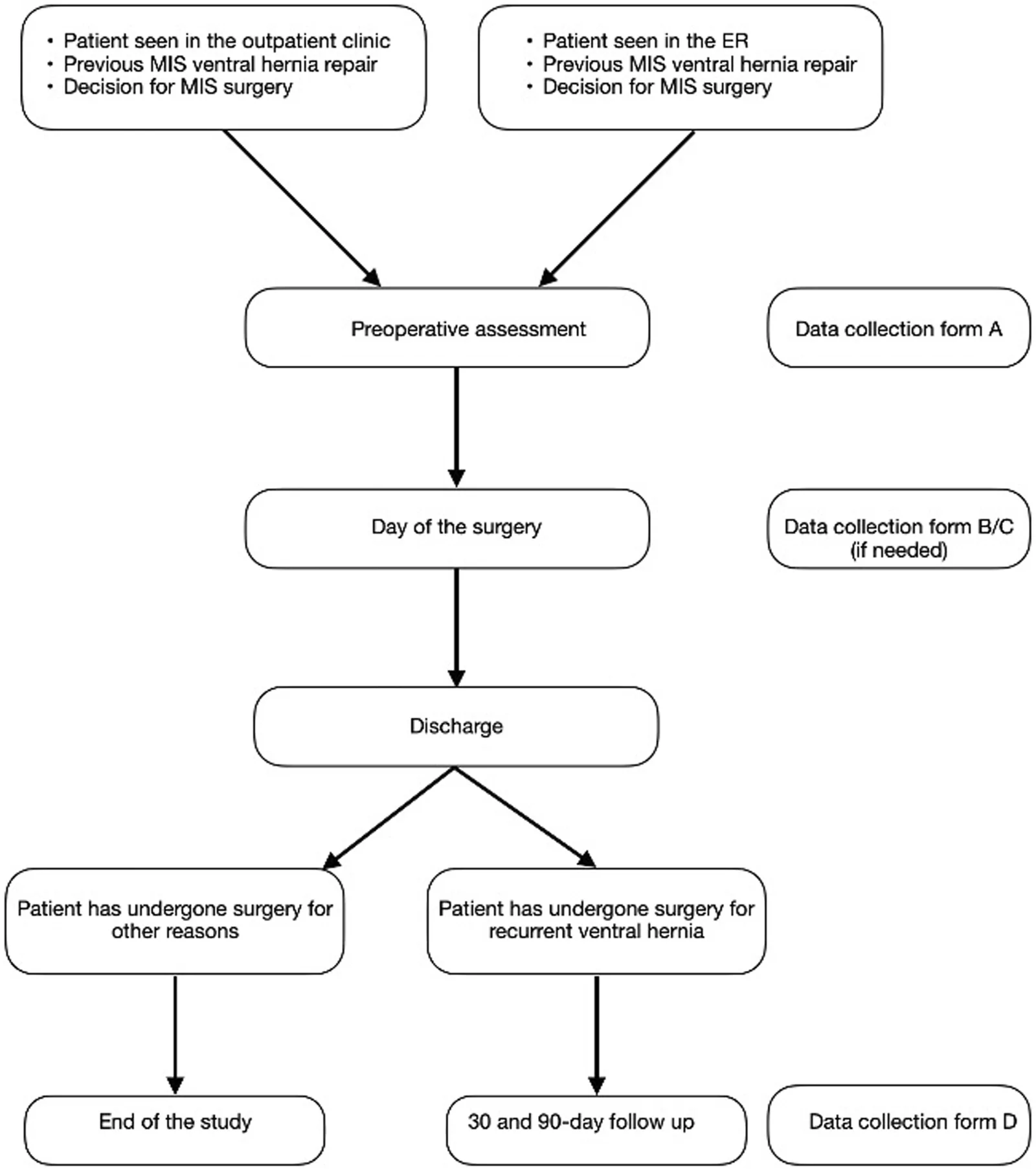
Study design of ORION study. MIS Minimally Invasive Surgery, ER Emergency Room.

Cohort definition. Two analysis cohorts are pre-specified:

– Cohort A–Non-hernia procedures: patients undergoing MIS for indications other than recurrent hernia repair (e.g., cholecystectomy, colorectal).
– Cohort B–Recurrent hernia repair: patients undergoing MIS to repair a recurrent ventral/incisional hernia after prior mesh placement.

Follow-up policy: Scheduled follow-up at 30 and 90 days applies only to Cohort B. Cohort A is assessed up to discharge (in-hospital outcomes only). We intentionally restricted scheduled post-discharge follow-up to recurrent hernia repairs to prevent indication-driven heterogeneity in outcome ascertainment. This preserves internal validity for early morbidity analyses while maintaining external validity for the primary intraoperative endpoint, which is captured across all MIS-after-mesh procedures.

## Methods

Participating centres will enroll consecutively eligible patients after local registration according to institutional policy. Each site designates a senior surgeon (local lead) as principal investigator, supported by one or two collaborators involved in perioperative care. Data are recorded prospectively through a dedicated web-based case report form (CRF) hosted on a GDPR-compliant platform. Sites will be recruited with the help of advertising from the European Hernia Society. Throughout the study period, all eligible surgeries will be recorded into the database in real time.

### Eligibility criteria

All adult patients (≥18 years) undergoing minimally-invasive surgery following previous minimally invasive ventral hernia repair (primary, recurrent or incisional) with intraperitoneal or extraperitoneal (either preperitoneal or retromuscular) mesh placement are eligible. Both elective and emergency cases are included to ensure real-world representation.

### Inclusion criteria

- Adult patients (18 years or older).
- Patients who have previously undergone minimally-invasive ventral hernia repair (primary, recurrent or incisional) using intraperitoneal or extraperitoneal (either preperitoneal or retromuscular) mesh placement.
- Patients undergoing scheduled or emergent minimally-invasive abdominal surgery for any reason (e.g., recurrent hernia repair or surgery for other abdominal pathologies).
- Patients who initially underwent a MIS abdominal procedure but required conversion to open surgery could be included, provided that an initial exploratory phase and attempt at adhesiolysis were performed.

### Exclusion criteria

- Patients scheduled for a primary open abdominal operation without an initial minimally invasive exploration
- Patients who have previously undergone an open ventral hernia repair
- No informed consent.

### Eligibility setting

Both elective and emergency procedures are eligible. *Emergency status (yes/no)* is recorded at baseline and reported in the main analysis. The primary statistical model will be stratified by emergency status, with a pre-specified sensitivity analysis excluding emergencies. An interaction term (*emergency × prior mesh position*) will be explored to evaluate potential effect modification.

### Primary outcome

- Adhesiolysis-related visceral injury during subsequent minimally invasive abdominal surgery. (Cohort A + B)
- Definition: The occurrence of intraoperative injury to visceral organs (including bowel, bladder, or solid organs) during adhesiolysis in patients with prior minimally-invasive intraperitoneal or extraperitoneal mesh placement. Omental injury or division is regarded as inherent to adhesiolysis and is not counted as a primary-outcome event; it is recorded separately as a descriptive intraoperative variable.
- Measurement: Recorded by the operating surgeon intraoperatively. Injuries will be categorized into:
o *Serosal tear*
o *Full-thickness bowel injury (enterotomy/perforation)*
o *Other visceral/solid-organ injury* (e.g., bladder, spleen, liver, stomach)

### Secondary outcomes

1. Incidence of Adhesion-Related Complications (Cohort A + B):

Definition: The occurrence of complications such as bowel obstruction, bowel perforation related to adhesions in patients who have undergone minimally-invasive intraperitoneal mesh versus extraperitoneal mesh placement.

Measurement: Documented through clinical follow-up, imaging, and operative findings during subsequent minimally-invasive abdominal procedures.

2. Duration of Adhesiolysis (Cohort A + B):

Definition: The time required (min) to perform adhesiolysis during subsequent surgery recorded intraoperatively as stated by the operating surgeon.

3. Conversion rate (Cohort A + B)

Definition: Declare if the MIS procedure is, at some point, converted to open surgery and the reason underneath this change (mesh adhesion related reason, other adhesion related reason, other reason)

4. Characterization of Postoperative Adhesion (Cohort A + B):

Definition: Adhesions are primarily attributed to the mesh, fixation devices (whether absorbable or non-absorbable), or unrelated factors not associated with the prior hernia repair surgery.

5. Adhesion Classification (Cohort A + B):

Definition: The severity and location of adhesions found during surgery, classified using the Peritoneal Adhesion Index (PAI). Furthermore, the abdomen will be mapped into five regions (RUQ, LUQ, RLQ, LLQ, Pelvis). Mono-quadrant adhesiolysis involves a single region; multi-quadrant adhesiolysis involves ≥2 regions. For each case, the number and identity of regions will be recorded, together with the estimated proportion of operative time dedicated to adhesiolysis (0%–25%–50%–75%–100%) and adhesion tenacity (flimsy/moderate/dense).

The following outcomes are assessed only in patients undergoing minimally invasive surgery for hernia recurrence (Cohort B):

6. Reason and Management of Recurrences (Cohort B):

Definition: The reason for hernia recurrence is defined as the most likely anatomical or technical mechanism leading to failure of the previous minimally invasive ventral hernia repair (e.g., Central mesh failure, marginal recurrence, mesh migration). The management is the surgical approach used to address hernia recurrence in patients with prior extraperitoneal or intraperitoneal minimally-invasive mesh placement.

Measurements will include:

- The approach used for managing recurrences (e.g., re-IPOM or mesh placement in other anatomical planes).
- Type of mesh used for the repeat surgery (same mesh type or different).
- Fixation methods employed in the repeat surgery (tack, suture, etc.).
- Complication rates following recurrent hernia repair (e.g., adhesion formation, bowel injury, infection).
  - Short-Term Postoperative Complications and Quality-of-Life (30 and 90-Day Follow-Up): The incidence of infection, pain, the rate of early hernia recurrence and reoperation within 30 and 90 days of surgery. Furthermore, Quality of Life will be recorded using the EuraHS-QoL scale at 30 and 90 days.

7. Neurological secondary outcomes (Cohort B).
  - Chronic post-surgical pain (CPSP) at 90 days, defined as an increase of ≥3 points in the Visual Analogue Scale (VAS) in the surgical area compared with the pre-operative baseline, new since surgery and not explained by specific complications (e.g., recurrence, infection, obstruction), in patients with prior extraperitoneal or intraperitoneal minimally-invasive mesh placement and without previous chronic pain diseases. For patients enrolled before a pre-operative baseline VAS became available, CPSP is defined as new pain in the surgical area with VAS ≥3 that is new since surgery.
  - Suspected neuropathic features at 30/90 days using the DN4 validated questionnaire in patients with prior extraperitoneal or intraperitoneal minimally-invasive mesh placement and without previous chronic neuropathic features
  - (allodynia)/hyperesthesia, in patients with prior extraperitoneal or intraperitoneal minimally-invasive mesh placement and without previous (allodynia)/hyperesthesia
  - Sensory change at 30/90 days (binary): patient-reported hypoesthesia/paresthesia around port sites or incisions in patients with prior extraperitoneal or intraperitoneal minimally-invasive mesh placement and without prior sensory change.
  - Analgesic profile: opioid use at day-30 (yes/no). Pain intensity will use the VAS already collected in ORION in patients with prior extraperitoneal or intraperitoneal minimally-invasive mesh placement.

### Data collection and timeline

- Patient Enrollment: Data collection will start with patient enrollment and operative details collected at the time of surgery.
- Time Points:
  1. Initial Data Collection: At the time of surgery and immediately post-surgery for the entire cohort (for intraoperative details, mesh type, adhesiolysis performed). For Cohort B, a pre-operative baseline pain score (VAS) is recorded at enrollment to allow calculation of the change in VAS (ΔVAS) at follow-up.
  2. 30 and 90-Day Follow-up: Assess short-term complications and postoperative pain only for the cohort of patients undergoing MIS surgery for hernia recurrence (VAS scale).
- Data Management: Data will be entered into a centralized, secure database accessible to all participating centers.

### Ethical considerations

This project adheres to the Declaration of Helsinki and European data-protection regulations. Local approval (audit or ethical clearance, as applicable) must be obtained before patient inclusion.

All data are fully de-identified before upload; no patient identifiers are transmitted to the coordinating team. The link between study ID and hospital identifiers is stored locally in an encrypted, password-protected file accessible only to the site lead.

Because ORION involves no deviation from standard clinical practice and collects data already generated during routine care, it qualifies as a minimal-risk observational study.

Patients will be provided with information about the study, and informed consent will be obtained for participation.

### Data quality assurance

The coordinating team continuously monitors data completeness and consistency. Automated algorithms within the CRF identify missing or out-of-range entries. Local investigators receive summary dashboards and are encouraged to finalise entries within 14 days of surgery. Retrospective data completion is permitted when necessary, with full traceability ensured by the system’s audit log. A defined audit schedule is applied: central automated CRF checks run continuously; the coordinating team reviews data completeness and provides feedback dashboards to each site every 3 months; and a formal data-quality audit of a random 10% sample of enrolled records is performed for each centre every 6 months or after every 20 patients enrolled at that centre, whichever comes first. Each audit includes source-data verification of the key variables (prior mesh position and the primary outcome of adhesiolysis-related visceral injury), with queries returned to the site lead for resolution within 14 days.

Centres achieving high completeness and accuracy are acknowledged as full collaborators in resulting publications.

### Data analysis

Continuous and sociodemographic variables will be reported as mean and standard deviation (SD) for normally distributed data, or as median and interquartile range (IQR) when appropriate. Categorical variables will be presented as absolute and relative frequencies. To compare continuous variables between intraperitoneal mesh group and extraperitoneal mesh group, the t-test will be applied, or the Wilcoxon rank-sum test in case of non-normally distributed data. Chi-square tests or Fisher’s exact test will be used to assess differences in categorical variables. Differences in the proportion of intraoperative injuries during adhesiolysis among patients with prior minimally invasive intraperitoneal or extraperitoneal mesh placement will be evaluated using a Z test. A log-binomial regression model (or Poisson regression with robust variance estimation) will be used to calculate the relative risk (RR) of intraoperative injury between the two surgical procedures. Adjusted estimates will be provided by including in the model all clinical and sociodemographic variables that differ between the two groups (p < 0.20).

Moreover, Chi-square test and two separate logistic regression models will be implemented to assess the association between surgical approach (intraperitoneal mesh vs. extraperitoneal mesh) and (i) chronic post-surgical pain (CPSP) at 90 days, and (ii) opioid use at day 30. All clinical and sociodemographic variables showing a between-group difference with p < 0.20 will be included as covariates. Odds ratios (OR) and corresponding 95% confidence intervals (95% CI) will be estimated.

In addition, chi-square test and two separate logistic regression models for repeated measures will be used to evaluate the association between surgical approach (intraperitoneal mesh vs. extraperitoneal mesh) and (i) suspected neuropathic features, and (ii) sensory changes over time.

### Sample size

A sample size of 345 patients per group, for a total of 690 participants, will provide 90% power to detect a 10% absolute difference in the proportion of adhesiolysis-related visceral injuries between groups, assuming an event rate of 25% in patients with prior intraperitoneal mesh placement and 15% in those with prior extraperitoneal mesh placement. The calculation is based on a two-sided Z-test for two independent proportions with unpooled variance and a significance level of 5% (α = 0.05). Because robust comparative estimates are limited, these assumptions are based on available reoperation data after laparoscopic ventral hernia repair and should be considered pragmatic [12, 15].

## Discussion

The optimal anatomical position for mesh placement—intraperitoneal versus extraperitoneal—remains one of the most debated questions in abdominal wall surgery and has yet to be definitively established [6, 9]. The ORION study has been designed to address it through a prospective, multicentre, observational framework endorsed by the European Hernia Society.

The primary rationale for this study stems from the long-term complications associated with intraperitoneal mesh placement. Adhesion formation between the mesh and adjacent visceral structures—particularly the bowel—is a recognised consequence of intraperitoneal repair [10, 11]. The ORION study addresses this gap by capturing intraoperative findings across both elective and emergency surgery, thereby providing a real-world picture of the consequences of prior intraperitoneal versus extraperitoneal mesh placement encountered at the time of subsequent abdominal surgery.

This accurate analysis will allow a meaningful comparison between the two mesh placement strategies, and may ultimately inform the selection of operative technique based on the anticipated risk of adhesion-related complications at reoperation [10–12].

The study design also reflects a deliberate effort to capture the neurological sequelae of ventral hernia repair, an area that has received comparatively little attention in the existing literature. Chronic post-surgical pain (CPSP) and neuropathic features following hernia repair represent an under-recognised source of long-term morbidity, and their association with mesh position, fixation method, and port-site trauma remains largely unexplored. By incorporating validated neurological outcome measures—including the DN4 questionnaire, sensory assessments, and analgesic profiling at 30 and 90 days—the ORION study is positioned to generate novel data on pain chronification in this patient population. These findings could have significant implications for the selection of mesh type and fixation strategy [6, 9], particularly in patients identified as being at higher risk of neuropathic sensitisation.

The ORION study is not without limitations. As an observational, non-randomised study, it is subject to the inherent confounding that characterises this study design. Differences in patient selection, operative technique, and centre-level expertise may influence outcomes independently of mesh position, as suggested by real-world registry data demonstrating substantial variability in surgical practice across institutions [9]. A further limitation is intrinsic to the sampling strategy: as patients are identified at a subsequent operation rather than at the index repair, the study has no denominator of all originally treated patients and cannot estimate absolute risks per index procedure. The reported relative risks are therefore conditional on reoperation and may be affected by selection bias, so the findings are best read as hypothesis-generating. Because extraperitoneal repair is a more recent technique, intraperitoneal cases will tend to have a longer interval since the index procedure, making time and surgical era potential confounders that will be treated as covariates. Finally, unblinded intraoperative ascertainment of the primary outcome may introduce detection bias, mitigated but not removed by standardised injury definitions. The absence of blinding and the potential for recall bias in neurological assessments must also be acknowledged. Finally, the study period of 12 months, while sufficient to achieve the planned sample size of 690 patients, may limit the detection of late complications that manifest beyond the 90-day follow-up window—particularly adhesion-related bowel obstruction, which may present years after the index procedure; likewise, the hernia recurrence rate captured at 30 and 90 days reflects early repair failure only and underestimates the true long-term recurrence rate [11, 12].

Despite these limitations, the ORION study represents a timely and clinically relevant contribution to the field. Its prospective, multicentre design, combined with a comprehensive outcome framework that encompasses intraoperative findings, postoperative complications, recurrence management, and neurological sequelae, positions it to generate high-quality evidence capable of informing future clinical guidelines. The findings are expected to support surgeons in selecting the most appropriate mesh placement strategy for individual patients, with the ultimate goal of reducing long-term morbidity and improving quality of life following ventral hernia repair.

## Data Availability

The original contributions presented in the study are included in the article/supplementary material, further inquiries can be directed to the corresponding author.
